# Assessment of a systematic approach for implementing novel medications in clinical practice: an observational study with dapagliflozin

**DOI:** 10.1007/s00228-024-03707-4

**Published:** 2024-06-10

**Authors:** Helena Norberg, Therese Andersson, Erik Håkansson, Karin Hellström Ängerud, Krister Lindmark

**Affiliations:** 1 Department of Medical and Translational Biology, Umeå University, S-901 87 Umeå, Sweden; 2 Department of Public Health and Clinical Medicine, Umeå University, Umeå, Sweden; 3 Department of Nursing, Umeå University, Umeå, Sweden; 4 Department of Clinical Sciences, Danderyd Hospital, Karolinska Institute, Stockholm, Sweden

**Keywords:** Systematic implementation, Healthcare quality improvement, Chronic disease management, Dapagliflozin

## Abstract

**Objective:**

To assess a systematic implementation approach for introducing dapagliflozin to individuals with heart failure and reduced ejection fraction in an outpatient clinical setting.

**Methods:**

Retrospective medical record data were analysed. All individuals diagnosed with heart failure who resided within the hospital catchment area and had visited cardiology or internal medicine department between 2010 and 2019 were screened by using the main inclusion criteria from the DAPA-HF trial. The effectiveness of the previously described seven-step systematic implementation approach was assessed by the proportion receiving information letter, dapagliflozin treatment, follow-ups at 2–12 weeks and 12 months post-dapagliflozin initiation, persistence on dapagliflozin, adverse events, and reasons for discontinuation.

**Results:**

Of the 2433 individuals, 352 met the main DAPA-HF trial criteria in step 2. After exclusions in steps 3 and 4, 191 individuals remained. Of these, 158 were invited for eligibility discussion in step 5, with 107 having received an information letter beforehand. In step 6, dapagliflozin was prescribed to 69 individuals, and in step 7, follow-ups were conducted with 56 individuals at 2–12 weeks and 62 individuals at 12 months. Sixty out of 69 persisted on dapagliflozin after 12 months. Adverse events were reported by nine individuals. Discontinuation was attributed to reasons such as urinary tract infections, genital or abdominal discomfort, and hypotension.

**Conclusion:**

The systematic introduction of dapagliflozin to heart failure patients was effective. Despite this, challenges in uniformly implementing procedures across patients were evident, emphasizing the necessity for a systematic implementation approach.

## Introduction

The escalating costs of drug treatments place a significant burden on healthcare resources [1]. Among OECD countries in 2021, the total spending on retail medications accounted for one-sixth of overall healthcare expenditure and corresponded to an average of US$614 per capita and rising [2]. Over the past two decades, Swedish expenditures for reimbursement drugs have surged by 74% from SEK 17.4 billion in 2002 to SEK 31.8 billion in 2022, and is estimated to increase to SEK 39.7 billion in 2026 [3]. This underscores the growing importance of allocating limited healthcare resources to patients and medications that offer cost-effective solutions.

National joint introduction of new medications is applied in Sweden to achieve an equal, cost-effective, and appropriate use of drugs in the whole country [4]. However, the implementation of novel medications often faces obstacles, with progress hindered by clinical inertia [5]. While national joint introductions and guidelines provide a framework, local physicians often bear the responsibility for implementation, a task made challenging by the need for comprehensive education on newly approved drugs—a need that is not consistently met.

The conventional implementation approach, akin to a hit-or-miss strategy, often falls short of effectively targeting eligible patients, particularly those with chronic conditions receiving care across primary and secondary settings [6–14]. Moreover, certain demographics, such as women, older, and fragile individuals, are frequently underrepresented in clinical trials due to stringent eligibility criteria, further worsening inequalities to access novel therapies [15].

To address these challenges, we have developed a systematic implementation approach [16]. This approach focuses on the systematic screening of eligible patients based on objective criteria and has been successfully applied in various therapeutic areas, including heart failure and ophthalmology [16–19]. In this study, we aimed to evaluate the effectiveness of the implementation effort involving the sodium-glucose cotransporter-2 (SGLT2) inhibitor dapagliflozin for individuals with heart failure and reduced ejection fraction when applying the systematic approach in an outpatient heart failure clinic.

## Methods

### Study population and selection process

**Fig. 1:**
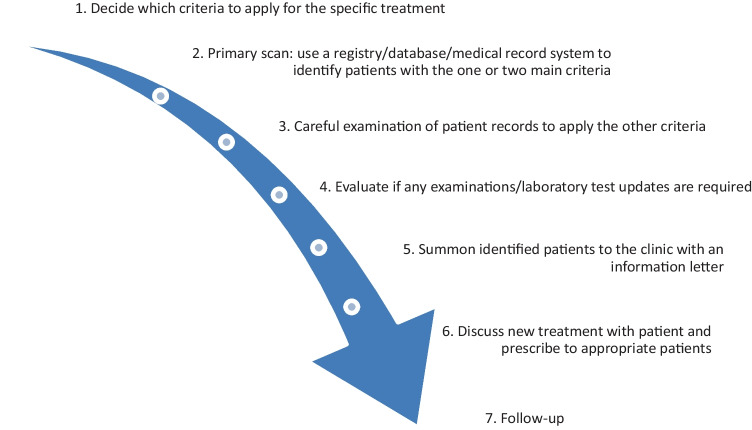
Overview of the workflow in the systematic implementation approach [16]. Published under the license of CC BY 4.0 (http://creativecommons.org/licenses/by/4.0/)

This is an observational study based on medical record data. A systematic implementation approach was used, that has been developed for implementation of novel medication in clinical practice. The approach was first developed with implementation of sacubitril-valsartan as a model [16]. The systematic approach consists of seven steps, from deciding implementation criteria (steg 1), screening of eligible individuals in available registries, databases, or medical records (step 2 and 3), to updated examinations and laboratory test (step 4), invitation with an information letter (step 5), discussion and initiation of the novel medication to appropriate individuals (step 6), and finally follow-up (step 7). An overview of the systematic implementation approach is shown in Fig. 1.

To evaluate how the systematic implementation approach function in clinical settings, we applied the approach for introduction of dapagliflozin at the Heart Centre, Umeå University Hospital, Sweden. The first two steps of the approach were performed in a previously published observational study, Håkansson et al. [18]. In brief, the study population consisted of all patients 18 years or above, with at least one visit at the Heart Centre or Department of Internal Medicine at Umeå University Hospital, between January 2010 and December 2019. Additionally, patients in the study population had a heart failure diagnosis (10th revision of the *International Classification of Disease and Related Health Problems* codes I50.X, I42.0, I46.6, I42.7, I42.9, I11.0, I13.0, I13.2), and were residents within the hospital catchment area. The hospital is the only hospital within the catchment area. Data was manually extracted from the hospital’s electronic medical record system (NCS Cross), according to a prespecified protocol to be able to perform the selection process.

In step 1, the selection of criteria was discussed and decided in accordance with cardiologists and nurses at the Heart Centre in order to choose the most clinically relevant criteria from the DAPA-HF trial [20], that also was accessible in medical record data [18]. The established criteria comprised five pivotal inclusion and exclusion criteria that were implemented in the DAPA-HF trial, namely: (i) left ventricle ejection fraction (LVEF) of 40% or less, (ii) NT-proBNP above 600 pg/ml, or at least 400 pg/ml, if the patient had been hospitalized for heart failure within the last 12 months. If the ECG showed atrial fibrillation or flutter at inclusion, the threshold was at least 900 pg/ml even if they had a recent hospitalization, and exclusion if (iii) estimated glomerular filtration rate (eGFR) of less than 30 ml/min/1.73 m^2^, (iv) symptoms of hypotension or a systolic blood pressure less than 95 mmHg, (v*)* type 1 diabetes mellitus [18].

In step 2, the above-described criteria were applied on the entire heart failure population to select eligible individuals for dapagliflozin. In steps 3 and 4, a more careful examination of medical records, as well as updated laboratory tests and examinations, such as echocardiography, were conducted closer to the planned outpatient visit to exclude individuals who had lost their eligibility since the primary selection.

In step 5, individuals identified as theoretical eligible were invited to an outpatient visit with an information letter, explaining why they were invited and a brief information about the new heart failure medication they were going to discuss with their physician. For individuals who already had a scheduled appointment at the heart failure outpatient clinic, their physician was allowed to choose whether the information letter would be sent in beforehand to the patient or not.

In step 6, individuals were prescribed dapagliflozin when the patient and physician agreed that it seemed to be a wise treatment option. In step 7, the physicians followed-up the initiated treatment within 2–12 weeks by telephone and after 12 months with an outpatient visit or by telephone. The follow-up focused on questions regarding heart failure symptoms, adverse events, and continuation of treatment.

The revised Lund-Malmö formula was used to estimate glomerular filtration rate (eGFR) instead of CKD-EPI and MDRD, as the revised Lund-Malmö formula has shown better accuracy in this heart failure population [21, 22].

### Outcome parameters

The following outcome parameters were used: (i) Proportion of patients receiving the information letter during practical implementation, (ii) proportion of intended patients receiving dapagliflozin, (iii) proportion of telephone follow-ups within 2–12 weeks after initiation of dapagliflozin, (iv) proportion of follow-ups 12 months after initiation of dapagliflozin, (v) proportion persistence on dapagliflozin after 12 months, (vi) frequency of adverse events, (vii) reasons for discontinuation, (vii) effects of treatment.

### Statistical analysis

In the selection process in step 2, each criterion was applied one by one. The outcome parameters were analysed with descriptive statistics. Continuous variables with normal distribution are presented as means with standard deviations, while non-normal distribution variables are displayed as medians with interquartile ranges. Categorical variables are summarized with frequencies and percentages. The SPSS version 27 was used for all the analyses.

## Results

**Fig. 2:**
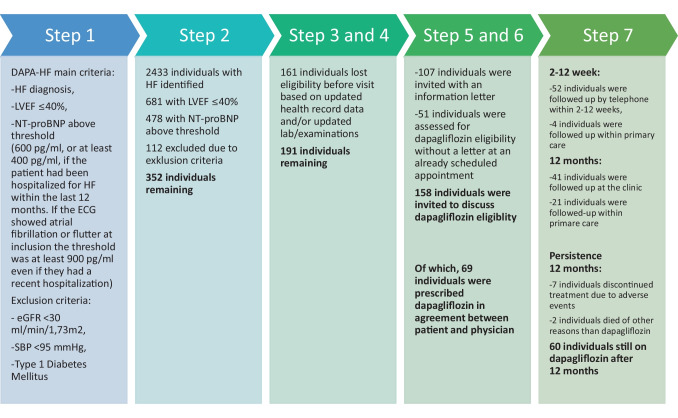
The selection process for introducing dapagliflozin to eligible patients with heart failure in an outpatient clinic, applying the seven-step systematic implementation approach with main entry criteria based on the DAPA-HF trial. eGFR, estimated glomerular filtration rate; HF, heart failure; LVEF, left ventricular ejection fraction; NT-proBNP, N-terminal pro-B-type natriuretic peptide; SBP, systolic blood pressure

The selection process and subsequent application of the systematic implementation approach are illustrated in Fig. 2. In step 2, when the main entry criteria of the DAPA-HF trial were applied to the heart failure population at Umeå University hospital, 352 out of 2433 individuals (14.5%) were identified eligible for dapagliflozin in the primary selection. Among individuals with left ventricular ejection fraction (LVEF) 40% or less, the most common criterion for exclusion was NT-proBNP level (*n* = 203), followed by eGFR (*n* = 83), systolic blood pressure (*n* = 26), and diabetes mellitus type 1 (*n* = 3).

Fourteen months after the primary selection in step 2, the third and fourth steps were applied. In total, 161 individuals lost their eligibility due to updated examination of medical records and/or laboratory test and examinations, resulting in 191 individuals remaining. The most common reasons for exclusion in these steps were that the individuals were deceased (*n* = 47), recovered ejection fraction (*n* = 30), dementia (*n* = 27), been already prescribed dapagliflozin (*n* = 15), living on a nursing home (with probable multiple comorbidities and/or cognitive disorders) (*n* = 14).

In step 5, the information letter was used to invite individuals to an outpatient visit in 107 out of 191 cases (56%). Out of the 84 individuals who did not get an information letter, 51 individuals already had a scheduled appointment at their cardiologist. In these cases, the cardiologist assessed the eligibility of dapagliflozin according to the predefined criteria at the next appointment. Ten individuals declined to attend the medical appointment after receiving the letter. The remaining 23 individuals did not receive a letter according to medical records. In summary, 158 out of 191 eligible individuals (82.5%) had a documented assessment for dapagliflozin in the medical records. Due to the Covid-19 pandemic, most of the outpatient appointments were performed via telephone.

In step 6, the patient and physician agreed to prescribe dapagliflozin for 69 out of the 191 theoretically eligible individuals (36%). Characteristics of these 69 individuals are presented in Table 1. Among the 122 individuals to whom dapagliflozin was not initiated within the study period, the most common reasons were individuals declining due to being asymptomatic (*n* = 38), LVEF or NT-proBNP had improved (*n* = 14), deceased before initiation (*n* = 11), other heart failure medications were prioritized (*n* = 10), multimorbidity (*n* = 9), low renal function (*n* = 7), hypotension (*n* = 5), high risk of acidosis (*n* = 3). On the other hand, 26 of these 122 individuals were initiated on a SGLT2 inhibitor at a later occasion, after the study period.

**Table 1:** Characteristics of eligible individuals with heart failure and reduced ejection fraction who fulfilled main criteria of the DAPA-HF trial and were initiated on dapagliflozin

**Characteristics**	**Eligible population (*n*** **= 69)**
Age, years	78.3 (± 10.8)
Female sex, *n* (%)	19 (27.5)
Body mass index, kg/m^2^	28.8 (± 5.6)
Systolic blood pressure, mmHg	126 (± 19)
Left ventricle ejection fraction, % (SD)	34 (± 6)
Serum potassium, mmol/L	4.4 (± 0.3)
NT-proBNP, ng/L, median (IQR)	1574 (853–2974)
eGFR, ml/min/1.73m^2^	42 (± 18)
NYHA class, no (%)^a^	
I	4 (5.8)
II	28 (40.5)
III	31 (44.9)
IV	3 (4.3)
**Medical history**	
Hypertension, no (%)	36 (52.2)
Diabetes mellitus type 2, *n* (%)	18 (26.1)
Atrial fibrillation or flutter, no (%)	45 (65.2)
Ischemic etiology, no (%)	42 (60.9)
CABG or valve surgery, no (%)	13 (18.8)
**Heart failure treatment**	
ACE inhibitor or ARB, no (%)	42 (60.9)
ARNI, no (%)	27 (39.1)
Beta blocker, no (%)	66 (95.7)
MRA, no (%)	54 (78.3)
Loop diuretic, no (%)	48 (69.9)
Digitalis, no (%)	14 (20.3)
ICD, no (%)	17 (24.6)
CRT, no (%)^b^	18 (26.1)

All values were reported as mean ± standard deviation unless otherwise indicated

*ACE* angiotensin converting enzyme, *ARB* angiotensin receptor blocker, *ARNI* angiotensin receptor blocker and neprilysin inhibitor, *CABG* Coronary artery bypass grafting, *CRT* cardiac resynchronization therapy, *eGFR* estimated glomerular filtration rate, *ICD* implantable cardioverter-defibrillator, *NT-proBNP* N-terminal pro-B-type natriuretic peptide, *MRA* mineralocorticoid receptor antagonist

^a^NYHA class available for 66/69 individuals

^b^Including one his-bundle pacing

The follow-up in step 7 was conducted by telephone within 2–12 weeks of dapagliflozin initiation in 56 out of 69 cases (81%). Thirteen individuals did not have a documented follow-up in their medical records. Nine individuals reported adverse events. An overview of early adverse events is provided in Table 2.

**Table 2:** Frequency of adverse events reported by the participants at 2–12-week follow-up

**Adverse events**	**Eligible population (*n*** **= 69)**
Hypotension	2
Fatigue	1
Constipation	1
Creatinine elevation/anemia	1
Pain in the legs	1
Abdominal discomfort, itching, and blurred vision	1
Hoarseness	1
Genital discomfort	1
**Total no. adverse events**	**9**

At 12 months, 62 out of 69 individuals (90%) underwent follow-up: 41 individuals at an outpatient visit and 21 individuals at a primary care centre. The seven remaining individuals did not have a documented follow-up. As per the medical records, adverse events were reported by three individuals at 12 months: two individuals experienced frequent urinary tract infections, while one individual reported a genital burning sensation.

In total, 60 out of 69 individuals (87%) persisted on treatment at 12 months. Seven individuals discontinued treatment during the first year due to adverse events, while two individuals died during the follow-up period from causes unrelated to dapagliflozin. Reasons for discontinuation of dapagliflozin (*n* = 7) are outlined in Table 3. The two individuals that reported frequent urinary tract infections decided to discontinue dapagliflozin in concordance with their physician at the 12 months of visit. The other discontinuations occurred earlier during the year. Table 4 provides a summary of the outcome parameters.

**Table 3:** Reasons for discontinuation of dapagliflozin within the first year

**Number**	**Participant**	**Reason for discontinuation**
**1**	79-year-old woman	Genital discomfort
**2**	57-year-old man	Hoarseness
**3**	78-year-old woman	Abdominal discomfort, itching, and blurred vision
**4**	74-year-old woman	Hypotension
**5**	82-year-old man	Leg pain
**6**	79-year-old man	Urinary tract infections
**7**	61-year-old woman	Urinary tract infections

**Table 4:** Summary of outcome parameters

**Outcomes**	**Proportion**
Proportion utilizing the information letter during practical implementation	107 out of 191 (56%)
Proportion of intended patients receiving dapagliflozin	69 out of 191 (36%)
Proportion of telephone follow-ups within 2–12 weeks after initiation of dapagliflozin	56 out of 69 (81%)
Proportion of follow-ups within 12 months after initiation of dapagliflozin	62 out of 69 (90%)
Proportion persistence on dapagliflozin after 12 months	60 out of 69 (87%)

The effects of dapagliflozin initiation were monitored regarding heart failure hospitalizations, changes in NYHA class, and self-reported well-being (Table 5). During the first 12 months following the initiation of dapagliflozin, 55 out of 69 individuals (80%) experienced no hospitalizations due to heart failure. At the 12-month follow-up, assessments of NYHA class and self-reported well-being were not documented for 41% and 45% of the individuals, respectively. NYHA class improved in 5 (7%) individuals, remained unchanged in 34 (49%), and worsened in 2 (3%). Self-reported well-being was noted as improved in 5 (7%) individuals, unchanged in 23 (33%), and worsened in 5 (7%).

**Table 5:** Effect outcomes during first 12 months after dapagliflozin initiation (*n* = 69)

**Characteristics**	**At 12 months**
**Number of HF hospitalizations last year, no (%)**	
0	55 (79.7)
1	5 (7.2)
2 or more	5 (7.2)
Missing	4 (5.8)
**NYHA class, no (%)**^a^	
Better	5 (7.2)
Unchanged	34 (49.3)
Worse	2 (2.9)
Missing	28 (40.6)
**Self-reported well-being, no (%)**^a^	
Better	5 (7.2)
Unchanged	23 (33.3)
Worse	5 (7.2)
Missing	36 (52.2)

^a^Change from initiation of treatment with dapagliflozin

## Discussion

The systematic implementation approach was effective at introducing dapagliflozin in an outpatient heart failure clinic. The approach could identify eligible patients within the hospital’s catchment area according to prespecified objective criteria, which shows its feasibility in real-world settings. Despite performing the implementation of dapagliflozin in a specialist clinic with efforts to simplify the systematic approach, not all patients who fulfilled the eligibility criteria received the information letter or were assessed for dapagliflozin. This accentuates that education or time for the threating physician is often insufficient in ordinary care settings, which motivates the need for a systematic approach to implement novel medications. By using the limited resources of healthcare and focus the implementation of novel treatments to the intended patients, the more cost-effective the implementation will be.

The systematic approach shows that it is possible to manage an implementation process by using objective criteria, based on national guidelines or clinical study criteria. The systematic approach can be used to both speed up or take a more cautious implementation strategy. For example, if the safety profile of the novel medication is uncertain in real-world settings, it is possible to conduct more strict criteria in the first round of implementation. This would identify eligible patients that are highly representative of the population included in clinical trials, where the strongest evidence is shown. However, for a treatment like dapagliflozin the safety profile is not a problem since the substance has been approved and used without any major safety concern in real-world diabetes mellitus type 2 (T2DM) populations for several years [23, 24]. Now when dapagliflozin is approved for a wide range of patients (including T2DM, heart failure and chronic kidney disease), the financial burden rises. At this stage, the systematic approach can be used to prioritize which patients should receive treatment with dapagliflozin based on budget allocations.

The systematic implementation approach was developed to introduce sacubitril-valsartan in the same clinical setting as the current study. In addition, the first two steps of the approach have been used to assess the real-world eligibility of sacubitril-valsartan, SGLT2 inhibitors, and faricimab before market authorization for budget planning [17–19]. In the sacubitril-valsartan study, the eligibility assessment was based on the strict main criteria from the PARADIGM-HF trial resulting in few eligible patients in the real-world population compared to the SGLT2 inhibitors and faricimab studies. This shows the consequence when clinical trials apply strict criteria, which in turn diminishes the external validity of the evidence since old and frail patients are rarely included in clinical trials.

To apply an implementation process across all patients in a clinic can be difficult [25]. In the current study, it became evident that not all physicians choose to send their patients the information letter. We attempted to simplify the approach by allowing this possibility for physicians who already had a scheduled appointment with eligible patients at the outpatient clinic. Consequently, many patients did not receive the information letter beforehand, depriving them of the opportunity to consider the option independently before their cardiologist appointment. In our earlier research, patients expressed appreciation for receiving the information letter in advance, as it allowed them time to consider the new treatment on their own terms before their scheduled appointment [16]. Furthermore, medical record data is not always complete, there can be some additional patients that received the letter even though it was not documented.

We also learned that even though all physicians at the outpatient clinic were involved in the systematic implementation approach with dapagliflozin, the knowledge of the drug may not be entirely spread since not all identified patients were assessed for dapagliflozin eligibility. Additionally, as the systematic approach was not the standard implementation procedure at the clinic, an initial increased workload could be observed to assess patients’ eligibility based on medical records and to assess and initiate dapagliflozin to patients not already listed at the clinic. This may suggest that the process can work more efficiently if the work is concentrated on a smaller team of interested physicians, nurses, and pharmacists.

The majority of the patients were followed-up within 3 and at 12 months after initiation of dapagliflozin (81% and 90%, respectively), which is an expected result in real-world settings. Since there was no documented follow-up for the remaining patients, it was not feasible to analyse the reasons behind the lack of follow-up in these cases. This information would be valuable to investigate further prior to the implementation of the next novel medication in the clinic.

Regarding safety outcomes, dapagliflozin was well tolerated in our population with few reported adverse events and 87% persistence on treatment after 12 months. Seven patients discontinued dapagliflozin due to various adverse events. The findings align with the outcomes of the phase III trials DAPA-HF and DELIVER [20, 26], suggesting that the safety characteristics observed with dapagliflozin could be applicable to our heart failure cohort.

Dapagliflozin has shown to be effective both in clinical trials and real-world populations [20, 26–29]. In our small real-world cohort, only one-fifth of the individuals experienced a heart failure hospitalization during the first year after dapagliflozin initiation. Despite the substantial amount of missing data on changes in NYHA class and self-reported well-being, the high proportion of individuals with unchanged NYHA class and well-being suggests disease stabilization. Given the progressive nature of heart failure, this stabilization, along with the low number of hospitalizations, is considered a beneficial outcome.

### Limitations

This study has several limitations. This was an observational study based on medical record data which may not be completely documented for all included individuals. We could have missed eligible patients who met the dapagliflozin criteria but with missing parameters in their medical records, resulting in exclusion. Furthermore, missing data can also have resulted in an underestimation of the number of individuals receiving the information letter or being followed-up after initiation.

The current study was not an explicit implementation study since we performed the primary selection of step 2 in the Håkansson et al. paper long time in advance. The gap between steps 2 and 3 was mainly caused by awaiting regulatory approval for dapagliflozin in heart failure. Approximately 14 months later, the rest of the systematic implementation approach was continued with step 3 and forward. This resulted in many excluded individuals (*n* = 161) in steps 3 and 4 due to lost eligibility in the meantime. It is possible that we missed to treat some individuals that were eligible 14 months earlier but due to disease progress later had deceased or were too ill to still fulfil eligibility. For future use of the systematic approach, a more continuous process would be recommended.

At the 12-month follow-up, nearly one-third of the individuals were monitored at their primary care centres. Unfortunately, we did not have access to the primary care medical records, resulting in missing data, particularly for the variables NYHA class and self-reported well-being. These variables are also influenced by various factors such as disease progression, comorbidities, and other heart failure medications in addition to dapagliflozin. Therefore, the effect outcomes should be interpreted with caution.

The single-centre study design reduces generalizability to other settings. However, we have applied the systematic approach in both other heart failure treatments and other diseases, such as ophthalmology, which indicates that the approach is flexible and can be adapted to other settings as well.

### Future research

Previous and current studies have demonstrated that the systematic implementation approach is applicable across various settings and adaptable to different medical treatments. However, further research is warranted to assess the cost-effectiveness of this approach in clinical practice. Additionally, exploring the systematic approach across a broader range of diagnoses and medications would be of interest. The core principle remains consistent: screening and identifying suitable patients based on predefined objective criteria to optimize the utilization of healthcare resources.

## Conclusions

The systematic approach effectively introduced dapagliflozin to heart failure patients in an outpatient clinical setting. This adaptable approach helped identify eligible patients based on specific criteria, demonstrating its practicality in real-world settings. Despite efforts to simplify the process, not all eligible patients received the information letter or underwent dapagliflozin assessment. This highlights the challenges in consistently applying procedures across all patients, even in specialized clinics, emphasizing the need for a systematic implementation approach.

## Data Availability

The datasets used and/or analyzed during the current study are available from the corresponding author on a reasonable request.
